# Eriodictyol Alleviated LPS/D-GalN-Induced Acute Liver Injury by Inhibiting Oxidative Stress and Cell Apoptosis via PI3K/AKT Signaling Pathway

**DOI:** 10.3390/nu15204349

**Published:** 2023-10-12

**Authors:** Xiaomei Zheng, Xinyan Wu, Qiqi Wen, Huaqiao Tang, Ling Zhao, Fei Shi, Yinglun Li, Zhongqiong Yin, Yuanfeng Zou, Xu Song, Lixia Li, Xinghong Zhao, Gang Ye

**Affiliations:** College of Veterinary Medicine, Sichuan Agricultural University, No. 211 Huimin Road, Wenjiang District, Chengdu 611130, China; 2021203001@stu.sicau.edu.cn (X.Z.); 2020203016@stu.sicau.edu.cn (X.W.); 13488975707@163.com (Q.W.); turtletang@163.com (H.T.); LingZhao@sicau.edu.cn (L.Z.); fei_shi@sicau.edu.cn (F.S.); liyinglun01@163.com (Y.L.); yinzhongq@163.com (Z.Y.); yuanfengzou@sicau.edu.cn (Y.Z.); songx@sicau.edu.cn (X.S.); lilixia905@163.com (L.L.); xinghong.zhao@sicau.edu.cn (X.Z.)

**Keywords:** eriodictyol, acute liver injury, network pharmacology, apoptosis, PI3K/AKT signaling pathway

## Abstract

Eriodictyol occurs naturally in a variety of fruits and vegetables, and has drawn significant attention for its potential health benefits. This study aims to look into the effects of eriodictyol on acute liver injury (ALI) induced by LPS/D-GalN and elucidate its potential molecular biological mechanisms. A total of 47 targets were predicted for the treatment of ALI with eriodictyol, and the PI3K/AKT signaling pathway played a key role in the anti-ALI processing of this drug. The in vivo experiment showed that eriodictyol can effectively reduce liver function-related biochemical indicators such as ALT, AST, and AKP. Eriodictyol can also up-regulate the levels of SOD and GSH, and inhibit the release of IL-1β, IL-6, and TNF-α. Additionally, TUNEL staining, immunohistochemistry, and RT-PCR experiments showed that eriodictyol activated the PI3K/AKT pathway and decreased the expression of Bax, caspase3, and caspase8 while increasing the expression of Bcl-2 m-RNA. Finally, molecular docking experiments and molecular dynamics simulations confirmed the stable binding between eriodictyol and PI3K, AKT molecules. This study showed that eriodictyol can activate the PI3K/AKT signaling pathway to alleviate ALI-related oxidative stress and apoptosis.

## 1. Introduction

Acute liver injury (ALI) is a pathological condition that arises from a variety of etiologies, such as sepsis-induced hepatotoxins, drug-related adverse events, alcohol overuse, metabolic syndrome, hepatitis viral infections, or bacterial infections that invade the liver [1]. The frequency of ALI cases is on the rise every year in China, and there is a risk that severe liver damage could potentially result in death or liver failure [2]. Despite strenuous efforts and attempts to explore therapeutic strategies beneficial to ALI, the effective and safe drugs are so discovering new medications that are effective in protecting the liver and have minimal side effects is highly important [3].

The diverse range of biological activities and desirable health benefits have recently made natural products more popular [4,5]. Eriodictyol, a flavonoid substance, is present in medicinal plants, vegetables, and citrus fruits, this compound is of potential significance to human health [6]. A wide array of pharmacological activities is exhibited by Eriodictyol, including antioxidant properties [7], anti-inflammatory effects [8], anti-tumor potential [9], and so on. Several studies have validated the ability of eriodictyol to mitigate acetaminophen-induced hepatotoxicity [10], as well as alleviate liver injury caused by arsenic trioxide through activating the Nrf2 pathway [11]. In addition, eriodictyol also showed protective effects on nervous system diseases, such as improving cognitive dysfunction [12]. Although eriodictyol is a strong antioxidant that may help eliminate free radicals and protect liver cells, its protective effects and mechanisms have not been fully studied.

In prior research, it was observed that the extract derived from *Swertia cincta* Burkill exhibits a specific protective effect on ALI. Notably, eriodictyol was identified as the primary active compound within the *Swertia cincta* Burkill extract [13]. This observation motivated us to further investigate and verify the liver-protective effects attributed to eriodictyol. Network pharmacology has risen worldwide in recent years, as a subfield of pharmacology, it offers a systematic approach to studying traditional Chinese medicine by establishing the connection between drugs, diseases, and syndromes. Network pharmacology has emerged as a prevalent research approach for investigating the molecular mechanisms of new drugs. The objective of this study was to explore the potential therapeutic targets and signaling pathways of eriodictyol in protecting against liver injury using network pharmacology. Subsequently, these findings were validated by establishing experimental models to assess the effects and mechanisms of eriodictyol on ALI in mice.

## 2. Materials and Methods

### 2.1. Chemicals and Reagents

Eriodictyol (≥98%) was provided by Nanjing Guangrun Biological Products Co., Ltd. (Nanjing, China). Silibinin (≥98%) and D-galactosamine (D-GalN) were provided by Shanghai Yuanye Biotechnology Co., Ltd. (Shanghai, China). Lipopolysaccharide (LPS) (055: B5) was bought from Sigma (Sigma, St. Louis, MO, USA). The assay kits for ALT, AST, AKP, γ-GT, CHE, TP, and ALB were supplied by Nanjing Jiancheng Bioengineering Institute (Nanjing, China), while SOD, MDA, and GSH were provided by Solarbio, (Beijing, China). The tunnel assay kit was purchased from Wuhan Servicebio, (Wuhan, China), and ELISA kits were purchased from FANKEWEI (Shanghai, China) used for measuring mice IL-6, IL-1β, and TNF-α.

### 2.2. Network Pharmacology

#### 2.2.1. Prediction of Eriodictyol Targets

In PubChem (http://pubchem.ncbi.nlm.nih.gov/ accessed on 3 January 2023), the SMILES code for the molecular structure of eriodictyol was obtained. The standard SMILES was then uploaded to the following databases: SwissTarget (http://swisstargetprediction.ch/ accessed on 4 January 2023), PharmaMapper (http://lilab-ecust.cn/pharmmapper/ accessed on 4 January 2023), Herb (http://herb.ac.cn/ accessed on 4 January 2023), ETCM (http://www.tcmip.cn/ETCM/ accessed on 4 January 2023), HIT (http://hit2.badd-cao.net/ accessed on 4 January 2023), and SEA (https://sea.bkslab.org/ accessed on 4 January 2023). These databases were utilized to identify the targets of eriodictyol. UniProt (http://www.uniprot.org/uniprot/ accessed on 4 January 2023) was employed to convert the target names into the official gene target symbol format.

#### 2.2.2. Predict Targets of ALI

Employing the search terms “acute liver injury”, “liver injury”, and “hepatic damage”, targets were predicted using the Genecard (https://www.genecards.org/ accessed on 5 January 2023), OMIM (https://www.omim.org/ accessed on 5 January 2023), TTD (http://db.idrblab.net/ttd/ accessed on 5 January 2023), PharmGKB (https://www.pharmgkb.org/ accessed on 5 January 2023), and DrugBank (https://go.drugbank.com/ accessed on 5 January 2023) databases. Subsequently, UniProt database was utilized to standardize the targets and verify their official gene names for accuracy.

#### 2.2.3. Eriodictyol-ALI Intersection Target

The transformed targets of eriodictyol and related to ALI were uploaded to the Draw Venn Diagram online platform at http://bioinformatics.psb.ugent.be/webtools/Venn/ (accessed on 6 January 2023). The resulting overlap of targets was then utilized to discern potential candidates for the treatment of ALI using eriodictyol.

#### 2.2.4. Construction of Protein Interaction (PPI) Network

Incorporate the shared targets of eriodictyol and ALI diseases into the STRING database (https://string-db.org/ accessed on 9 January 2023). In the interface, constrain the species to “*Homo sapiens*” and set the confidence score threshold to >0.4 for online PPI network construction. In the Export section, opt for the tsv file format for downloading and saving. Import this file into Cytoscape (https://cytoscape.org/ accessed on 9 January 2023) for visualization purposes. Utilize the network analyzer (http://apps.cytoscape.orgnetworkanalyzer accessed on 9 January 2023) to evaluate degree distribution, clustering coefficient, and edge centrality. These analyses will yield the final network graph and provide an initial insight into the core targets.

#### 2.2.5. GO, KEGG, and DO Analysis

Utilizing the Database for Annotation, Visualization, and Integrated Discovery (DAVID) (https://david.ncifcrf.gov/ accessed on 10 January 2023), we performed Disease Ontology (DO) analysis, Gene Ontology (GO) enrichment analysis, and Kyoto Encyclopedia of Genes and Genomes (KEGG) pathway analysis to assess the shared targets, employing criteria of FDR < 0.05 and *p* < 0.05. Additionally, bubble charts were constructed utilizing online resources available at http://www.bioinformatics.com.cn/ (accessed on 10 January 2023). R software (Version 4.1.1) was employed for visualizing the outcomes.

### 2.3. Evaluating Expression Patterns of AKT1 and PI3K

The Human eFP (Electronic Fluorescence Pictogram) browser, accessible at http://bar.utoronto.ca/efp_human/cgi-bin/efpWeb.cgi (accessed on 20 February 2023), offers additional details regarding prospective candidate genes [14]. In the study of gene expression profile, select the data source “Skeletal Immune Digestive”, select the “Absolute” mode, input the gene symbols “AKT1”, and “PIK3R1” in turn, and click “Go”. To quickly determine the level of expression of a given gene in liver tissue, generate an expression “analysis diagram” by coloring the human sample representation based on the gene of interest’s expression level. The diagram utilizes a yellow-red scale to describe the expression level.

### 2.4. Molecular Docking

The crystal structures of the PI3K (ID: 5DXT) and AKT (ID: 3QKK) receptors were retrieved from the Protein Data Bank (PDB) website (https://www.rcsb.org/ accessed on 16 January 2023). The three-dimensional configuration of the eriodictyol ligand was obtained from the PubChem database. AutoDockTools (Version 1.5.6) software was used to prepare the protein and ligand structures for docking, including the addition of charges and hydrogen atoms, as well as setting up the binding site. Molecular docking simulations were carried out using AutoDockTools, and the process was repeated three times to enhance the reliability of the results. The results were analyzed by determining the active site positions and calculating the binding energy and hydrogen bond numbers. A negative binding energy indicates the ligand’s ability to spontaneously bind to the receptor, while a lower binding energy value signifies a stronger binding affinity. Ligplot+ software (version 2.2.4) (http://www.ebi.ac.uk/thornton-srv/software/LIGPLOT/ accessed on 20 January 2023) and PyMOL (Version 4.6.0) software were employed for visualizing and examining the ligand-receptor interactions, facilitating the identification of crucial residues involved in the binding process.

### 2.5. Molecular Dynamics Simulation

ROMACS (version 2020.6) was utilized to conduct molecular dynamics (MD) simulations. The CHARMM36 force field, along with the TIP3P water model, was employed to model interactions. The simulation system comprised a solvation box with a cubic shape, where the edge length was set to 1.2. Periodic boundary conditions of the adjoint type were applied for a duration of 1 ns. Following solvation, ion equilibration was performed to attain an ion concentration of 0.145 M, simulating the human environment and achieving initial conformational equilibration. Subsequently, the equilibration phase was executed in two steps: first, 100 ps in the ensemble of constant particle number, volume, and temperature, followed by 100 ps in the ensemble of constant number, pressure, and temperature. The temperature and pressure were adjusted to 310 K and 1 bar, respectively. Finally, a 100 ns MD simulation encompassing the entire system was carried out. The nonbonded interaction cut-off value was set at 1.2 nm, and the PME algorithm was employed to calculate long-range electrostatic interactions. A time step of 2 fs was utilized, and the conformations were saved at intervals of 10 ps.

### 2.6. Animals and Experimental Design

In preparation for drug administration, eriodictyol was accurately weighed and dissolved in PBS to obtain concentrations of 1, 2, and 4 mg/mL. Silibinin was dissolved in PBS to achieve a concentration of 10 mg/mL. The dosage and administration method for eriodictyol were determined based on previous investigations [10,15].

Sixty male ICR mice (20 ± 2 g), aged 4 weeks, were obtained from Chengdu Dassy Biotechnology Co., Ltd. The mice were housed in a controlled environment with a constant temperature of (23 ± 2 °C), relative humidity of (55 ± 5%), and a 12-h light-dark cycle. They had *ad libitum* access to food and water. After a 7-day acclimatization period, the mice were randomly divided into six experimental groups as follows: (1): The control group (*n* = 10) received an intraperitoneal injection of physiological saline. (2) The LPS/D-GalN group (ALI model, *n* = 10) received an intraperitoneal injection of physiological saline. (3) The LPS/D-GalN+eriodictyol low-dose treatment group (eriodictyol 10 mg/kg, *n* = 10) received an intraperitoneal injection of eriodictyol at a dose of 10 mg/kg. (4) The LPS/D-GalN+eriodictyol medium-dose treatment group (eriodictyol 20 mg/kg, *n* = 10) received an intraperitoneal injection of eriodictyol at a dose of 20 mg/kg. (5) The LPS/D-GalN+eriodictyol high-dose treatment group (eriodictyol 40 mg/kg, *n* = 10) received an intraperitoneal injection of eriodictyol at a dose of 40 mg/kg. (6) The LPS/D-GalN+silibinin treatment group (silibinin 100 mg/kg, *n* = 10) received an intraperitoneal injection of silibinin at a dose of 100 mg/kg.

The administration was carried out once a day for a duration of 7 days. On the 7th day, mice in the LPS/D-GalN, LPS/D-GallN+eriodictyol (10, 20, and 40 mg/kg), and LPS/D-GalN+silibinin groups were intraperitoneally injected with 600 mg/kg of D-GalN and 10 μg/kg of LPS, following one hour after the administration of eriodictyol, silibinin, or physiological saline. The control group received a saline injection alone. After 6 h, all mice were euthanized to collect blood and tissue samples. Plasma was collected, followed by centrifugation at 3500× *g* for 10 min at 4 °C to obtain the supernatant, which was then stored at −80 °C.

### 2.7. Organ Index

The mice’s liver was obtained and weighed to determine the organ index using the formula: the weight of the organ (in grams) was divided by the body weight (in grams) and multiplied by 100%.

### 2.8. Biochemistry Analysis

The blood was centrifugated (3500× *g*, 10 min, 4 °C) and the supernatant was collected for analysis. The levels of alanine aminotransferase (ALT), aspartate aminotransferase (AST), albumin (ALB), total proteins (TP), alkaline phosphatase (AKP), γ-glutamyltransferase (γ-GT), and cholinesterase (CHE) in the serum were determined using assay kits specific to ALT, AST, ALB, TP, AKP, γ-GT, and CHE.

### 2.9. Hematoxylin and Eosin (H&E) Staining

The liver tissues of the mice were fixed in a solution of 4% paraformaldehyde. Subsequently, the tissues were embedded in paraffin to enable sectioning. The evaluation of histopathological changes was conducted through hematoxylin-eosin staining. The assessment of necrosis and inflammation in the liver was performed at a magnification of 400×. The most representative image depicting liver morphology for each group was photted and presented.

### 2.10. TdT-Mediated dUTP Nick End Labelling (TUNEL) Staining Analysis

Liver slices from different groups were subjected to dewaxing and rehydration. Following PBS washing, the slices were exposed to protease K at 37 °C for 30 min and then quenched with 3% hydrogen peroxide at room temperature for 10 min. After further PBS cleaning, the slices were incubated in a mixture of TUNEL labeling and enzyme solution at 37 °C for 1 h. To eliminate potential false positive results, two slices were also incubated in the labeling solution. Subsequently, the slices were treated with DAPI at room temperature for 5 min. TUNEL-positive cells were quantified under a microscope in three randomly non-adjacent areas at 400× magnification.

### 2.11. Analysis of the Antioxidant System

The SOD, GSH, and MDA assays, which are oxidative stress indicators, were conducted using the SOD, MDA, and GSH Detection Kit, following the instructions provided in the operation manual.

### 2.12. Detection of Anti-Inflammatory Biomarkers

Levels of interleukin (IL-6, IL-1β) and tumor necrosis factor α (TNF- α) in liver tissue were quantified using a commercial ELISA kit, following the manufacturer’s instructions.

### 2.13. Immunohistochemistry

The protein expression of PI3K and AKT in mouse liver tissues was detected via immunohistochemistry in mice liver sections by using standard immunohistochemistry methods [16]. The antibodies of PI3K (Cat No. 20584-1-AP) and AKT (Cat No. 60203-2-Ig) have been used in this study.

### 2.14. Quantitative Real-Time PCR (RT-PCR)

Total RNA was extracted from liver tissue using Trizol reagent (Transgene, Beijing, China), followed by performing a measurement of the concentration of total RNA in the sample. Subsequently, synthesis of cDNA was accomplished utilizing a High capacity RNA to cDNA kit (Transgene, Beijing, China).

The expression of the indicated genes was analyzed using RT-PCR amplified with SYBR Green (Transgene, Beijing, China). QX400 (Sichuan Jielaimei Technology Co., Ltd., Chengdu, China) was used for qPCR. A list of primer sequences is presented in Table 1. The relative levels of each expression were determined using the 2−ΔΔCq method, with GAPDH serving as the internal normalized reference gene. There are forward and reverse primer sequences listed in Table 1.

### 2.15. Statistical Analysis

The data were reported as the mean ± SEM and statistical analyses were performed using GraphPad Prism 9.0 software (GraphPad Software, La Jolla, CA, USA). Both *t*-test and ANOVA were utilized as statistical assessments. The significance level was established at *p* < 0.05.

## 3. Results

### 3.1. Identify the Potential Target of Eriodictyol in the Treatment of ALI

**Figure 1:**
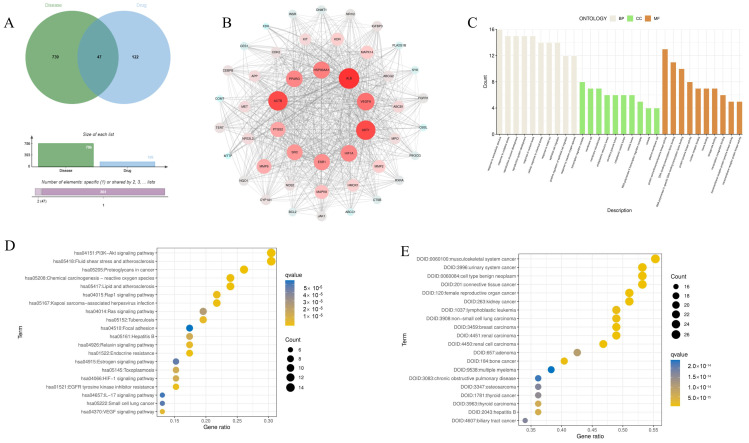
Network pharmacology. (**A**) Venn diagram shows the intersection target of eriodictyol and ALI, and the overlapping gene is considered as the potential therapeutic target of eriodictyol in treating ALI. (**B**) PPI network, a PPI network with 47 eriodictyol-ALI intersection targets. (**C**) GO enrichment analysis, including BP, CC and MF analysis. (**D**) Enrichment analysis of KEGG pathway of 47 presumptive targets. (**E**) DO enrichment analysis.

Through database analysis, a total of 169 targets for eriodictyol and 786 disease targets were identified. Additionally, 47 potential targets for the treatment of ALI with eriodictyol were pinpointed (Figure 1A).

### 3.2. PPI Network Construction and Analysis

The PPI network is shown in Figure 1B. Based on the Degree metric, which measured the connectivity, the top 10 targets including ALB, ACTB, AKT1, VEGF, and others were selected (Table 2). These targets hold substantial relevance in the medicinal context of eriodictyol for the treatment of ALI.

### 3.3. GO Function, KEGG Pathway Enrichment, and Disease Enrichment Analysis

The GO enrichment analysis conducted in this study focuses on three domains: cell components (CC), molecular functions (MF), and biological processes (BP) as outlined by Zheng et al. in 2020. Figure 1C displays the top 10 results for each domain (BP, CC, and MF). The results reveal that the biological processes primarily involve responses to oxidative stress (GO:0009991), reactions to extracellular stimulation (GO:0006979), and the development of reproductive structures (GO:0048608). Regarding cell components, the analysis identifies transcription regulator complexes (GO:0005667), membrane rafts (GO:0045121), and membrane microdomains (GO:0098857). In terms of molecular functions, the identified functions include protein serine/threonine/tyrosine kinase activity (GO:0004712), DNA binding transcription factor binding (GO:0140297), and RNA polymerase II-specific DNA-binding transcription factor binding (GO:0061629), among others.

Among the initial set of 20 enriched KEGG pathways, the “PI3K-Akt signaling pathway” has been recognized as a crucial pathway for the treatment of ALI using eriodictyol. The KEGG enrichment pathway map, illustrating the pertinent details of the 20 enriched pathways, was acquired through importation into “WeChat” (Figure 1D). The enrichment results related to diseases (Figure 1E) reveal that hepatitis B predominantly constitutes one of the diseases displaying potential target effects.

### 3.4. Expression Patterns of AKT1 and PI3K Genes in Liver Tissue

**Figure 2:**
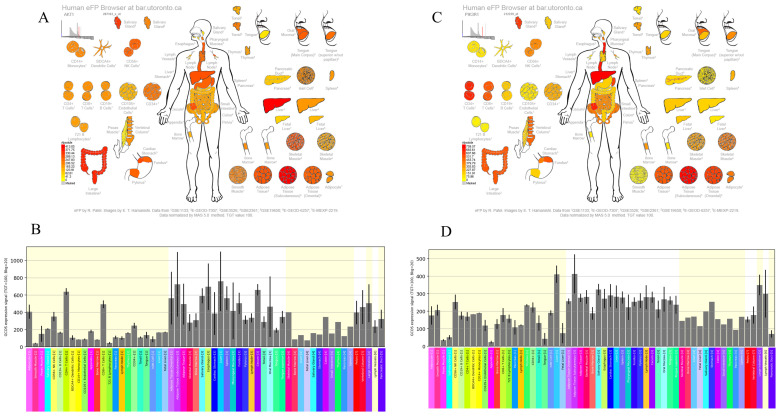
Illustrate the expression of AKT1 and PI3K in the human immune system using the human eFP browser. (**A**,**C**) present an anatomical map displaying the expression patterns, while (**B**,**D**) show the corresponding histograms for AKT1 and PI3K expression levels in the human immune system.

In the analysis, we examined the gene expression profiles of AKT1 and PI3K using the human eFP browser, which provides an overview of gene expression levels in the liver. The analysis shows that AKT1 and PI3K are highly expressed in the liver (Figure 2), indicating that the overexpression of AKT1 and PI3K genes may contribute to the recovery of liver injury.

### 3.5. Eriodictyol Alleviated LPS/D-GalN-Induced Liver Injury in Mice

**Figure 3:**
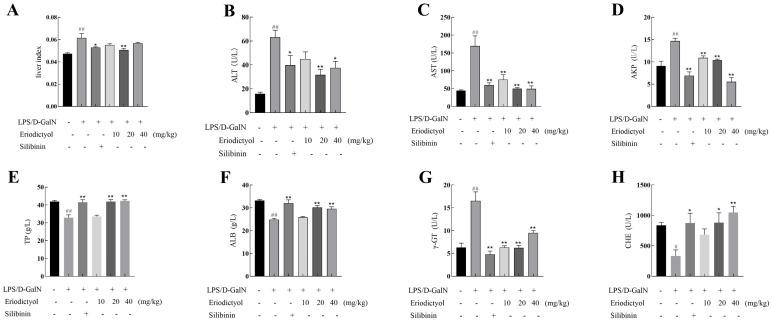
Eriodictyol mitigated ALI induced by LPS/D-GalN in mice. (**A**) Liver index, the contents of ALT (**B**), AST (**C**), AKP (**D**), TP (**E**), ALB (**F**), γ-GT (**G**), and CHE (**H**) in mice serum. The data, expressed as mean ± SEM (*n* = 6), revealed statistically significant differences. # *p* < 0.05 and ## *p* < 0.01 compared to the control group; * *p* < 0.05 and ** *p* < 0.01 compared to the model group.

Liver index, ALT, AST, AKP, TP, ALB, γ-GT, and CHE levels were assayed to assess the impact of eriodictyol on ALI. In comparison to the control group, the liver index (Figure 3A) exhibited a significant increase (*p* < 0.01) in mice treated with LPS/D-GalN. However, when compared to the model group, the positive control silibinin group and the medium dose group of eriodictyol demonstrated a significant reduction effect on the liver index. The results also indicated that LPS/D-GalN-induced significantly increased the levels of ALI, ALT, AST, AKP, and γ- GT, while decreasing TP, ALB, and CHE levels significantly (Figure 3B–H). However, compared to the corresponding levels in the model group, the therapeutic administration of silibinin and eriodictyol significantly reverses the liver injury indicators induced by LPS/D-GalN.

### 3.6. Eriodictyol Improves Liver Pathology and Apoptosis in Mice Induced by LPS/D-GalN

**Figure 4:**
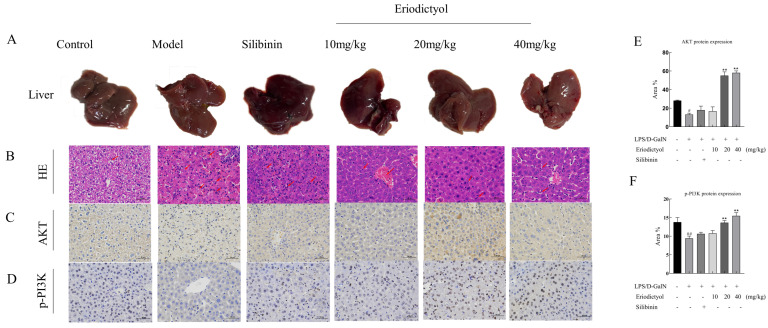
H&E staining and Immunohistochemistry. (**A**) Liver appearance. (**B**) Histopathological analysis of liver tissues (H&E, 400×). (**C**,**D**) Immunohistochemical analysis was conducted to assess the expression levels of AKT and p-PI3K in liver tissue (IHC, 400×). (**E**,**F**) Histograms show AKT and p-PI3K immunohistochemistry, expressed in region%. Scale, 50 μm. The data, expressed as mean ± SEM (*n* = 6), revealed statistically significant differences. # *p* < 0.05 and ## *p* < 0.01 compared to the control group; ** *p* < 0.01 compared to the model group.

**Figure 5:**
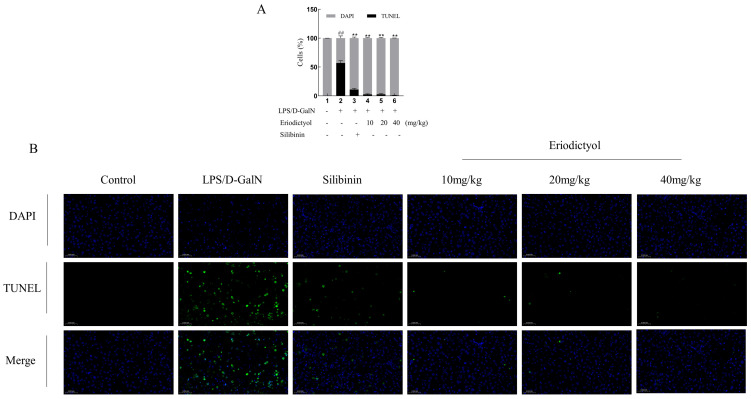
TUNEL histology. (**A**) The calculated percent TUNEL-positives cells. (**B**) The representative TUNEL stain images (400×). DAPI-positive cells were identified by blue fluorescence, while TUNEL-positive cells were indicated by green fluorescence. Scale, 50 μm. The data, expressed as mean ± SEM (*n* = 6), revealed statistically significant differences. ## *p* < 0.01 compared to the control group; ** *p* < 0.01 compared to the model group.

To further validate the hepatoprotective effect of eriodictyol, a histopathological examination was conducted (Figure 4A,B). The model group exhibited a substantial presence of hepatocyte necrosis, nuclear fragmentation, dissolution, and cytoplasmic disintegration. Additionally, there was a noticeable infiltration of neutrophils and a scattered distribution of Kupffer cells within the liver tissue. Furthermore, there was extensive hepatic sinus congestion and expansion. In contrast, in the control group, no evident necrosis or infiltration of inflammatory cells was observed in the control group. These changes in the model group were improved after treatment with silibinin and eriodictyol, these findings further confirmed the hepatoprotective effect of eriodictyol against LPS/D-GalN-induced ALI. Subsequently, we examined hepatocyte apoptosis in the context of ALI induced by LPS/D-GalN. Immunohistochemistry staining was conducted on mouse liver tissue to assess the expression of phosphorylated PI3K and AKT (Figure 4C–F). The brownish-yellow particles in the picture are positive expressions of the two proteins. The AKT and p-PI3K immunohistochemical histogram showed a significant decrease in the expression of p-PI3K and AKT was observed in the liver tissue of mice in the model group (*p* < 0.05) when compared to the control group. Conversely, treatment with eriodictyol resulted in a significant increase in the expression of p-PI3K and AKT in the liver tissue of mice compared to the model group (*p* < 0.05). TUNEL staining revealed a significant increase (*p* < 0.01) in the population of apoptotic cells in the liver tissue of the model group compared to the control group (Figure 5), thereby promoting hepatocyte death. However, eriodictyol effectively attenuated hepatocyte death induced by LPS/D-GalN. In comparison to the model group, the treatment group exhibited a notable reduction in TUNEL-positive staining, indicating a decrease in liver tissue apoptosis.

### 3.7. Eriodictyol Mitigated the Oxidative Stress and Inflammation Induced by LPS/D-GalN

**Figure 6:**
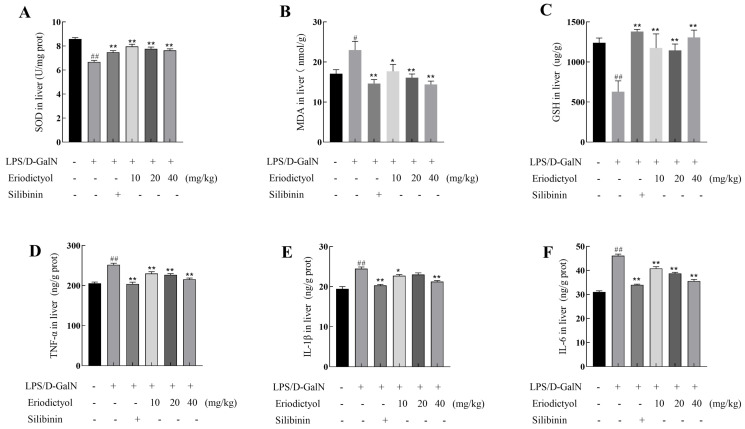
The impact of eriodictyol on oxidative stress and inflammation was evaluated by measuring the levels of SOD (**A**), MDA (**B**), and GSH (**C**) in liver tissue of mice. Additionally, the concentrations of TNF-α (**D**), IL-1β (**E**), and IL-6 (**F**) in liver tissue of mice were also assessed. The data, expressed as mean ± SEM (*n* = 6), revealed statistically significant differences. # *p* < 0.05 and ## *p* < 0.01 compared to the control group; * *p* < 0.05 and ** *p* < 0.01 compared to the model group.

As depicted in Figure 6A–C, the model group exhibited a substantial elevation in MDA content (*p* < 0.05) and a significant decrease in SOD and GSH activities (*p* < 0.01) compared to the control group. These findings indicated the induction of robust oxidative stress in mice induced by LPS/D-GalN. Eriodictyol treatment can decrease oxidative stress in a dose-dependent manner, by down-regulating the release of MDA in comparison to the model group. Additionally, all doses of eriodictyol substantially elevated the expression of SOD and GSH. These findings suggested that eriodictyol administration alleviates ALI induced by LPS/D-GalN by enhancing the synthesis of antioxidant enzymes and suppressing lipid peroxidation. As illustrated in Figure 6D–F, the levels of IL-6, IL-1β, and TNF-α were significantly elevated in mice subjected to LPS/D-GalN induction (*p* < 0.01), demonstrating the occurrence of pronounced liver inflammation. In comparison to the model group, the eriodictyol treatment group exhibited a significant reduction in IL-6 (Figure 6F), IL-1β (Figure 6E), and TNF-α (Figure 6D), with a dose-dependent decrease observed in IL-6 and TNF-α levels. Notably, the high dose of eriodictyol demonstrated similar effectiveness to the positive control, silibinin group. These findings highlight the anti-inflammatory properties of eriodictyol in LPS/D-GalN-induced ALI.

### 3.8. Eriodictyol Alleviates LPS/D-GalN-Induced ALI in Mice through Modulation of the PI3K/AKT Signaling Pathway and the Apoptosis Pathway

**Figure 7:**
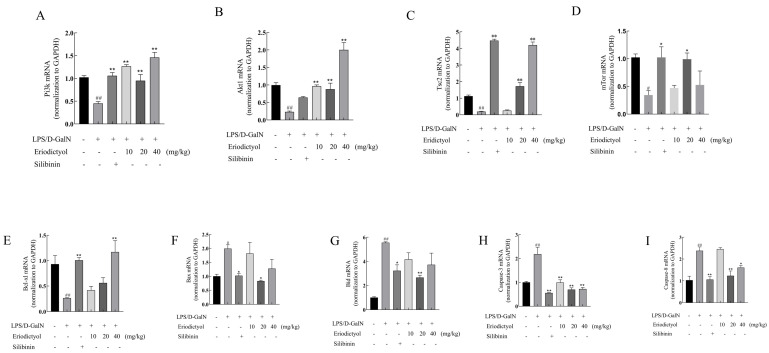
The impact of eriodictyol on the activation of the PI3K/AKT signaling pathway and apoptosis in liver tissues was investigated. The mRNA expression of Pi3k, Akt1, Tsc2, and mTOR was analyzed using RT-PCR (**A**–**D**). (**E**–**I**) RT-PCR analysis for the mRNA expression of Bcl-xl, Bax, Bid, Caspase-3, and Caspase-8. The data, expressed as mean ± SEM (*n* = 6), revealed statistically significant differences. # *p* < 0.05 and ## *p* < 0.01 compared to the control group; * *p* < 0.05 and ** *p* < 0.01 compared to the model group.

Figure 7A–D illustrates the mRNA expression of PI3K, AKT, TSC2, and mTOR were decreased in the model group when compared to the control group (*p* < 0.05). Conversely, both eriodictyol and silibinin substantially enhanced the mRNA expression levels of PI3K, AKT, TSC2, and mTOR of ALI mice (*p* < 0.05). These findings suggest that the hepatoprotective effect of eriodictyol may be attributed to the activation of the PI3K/AKT signaling pathway. Moreover, both the eriodictyol and silibinin groups exhibited a significant increase in Bcl-xl mRNA expression while decreasing the mRNA expression of Bax, Bid, Caspase-8, and Caspase-3 (Figure 7E–I).

### 3.9. Molecular Docking Verification

**Figure 8:**
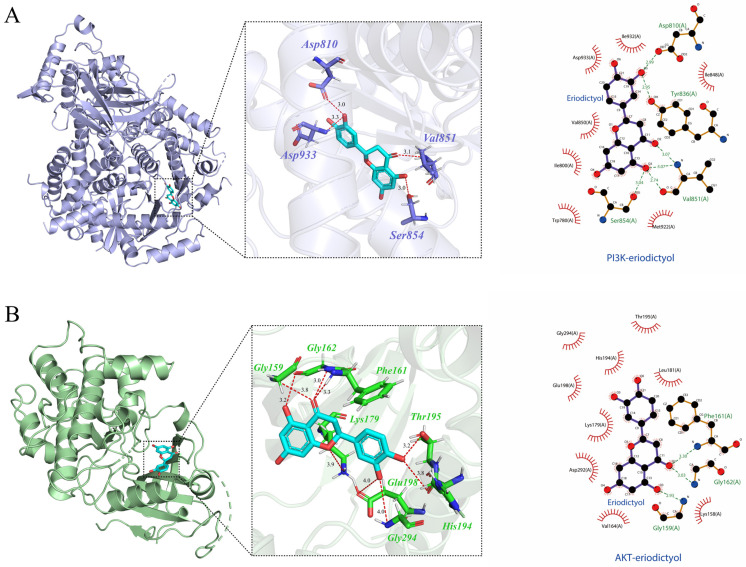
Molecular docking analysis. The binding mode of the eriodictyol-PI3K complex (**A**) and the eriodictyol-AKT composite (**B**) (3D and 2D images). The results represent three independent experiments.

Molecular docking analysis showed that eriodictyol can bind to the active pockets of PI3K and AKT crystal structures, and the overall binding conformation can be observed in the 3D diagram. The 2D diagram depicts the formation of robust hydrogen bonds between eriodictyol and specific amino acid residues of PI3K (Figure 8A), namely Ser854, Val851, Asp810, and Tyr836. Similarly, eriodictyol establishes steady hydrogen bonds with amino acid residues Gly159, Gly162, and Phe161 of AKT (Figure 8B). An activation energy below zero indicates that ligand and receptor can spontaneously bind, with a smaller value indicating stronger binding [17]. The binding energy of eriodictyol to PI3K and AKT is −8.147 kcal/mol and −9.021 kcal/mol. It is plausible to conclude that the ligand and the receptor establish a relatively stable complex, and can be further examined through subsequent kinetic verification.

### 3.10. MD Simulation to Explore the Interaction of Eriodictyol with PI3K and AKT

**Figure 9:**
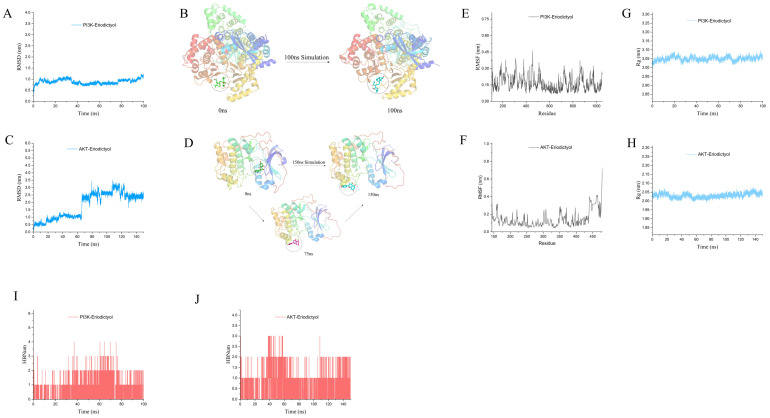
Molecular dynamics simulation. (**A**) RMSD of PI3K-eriodictyol complex in MD analysis. (**B**) Conformation of PI3K-eriodicityol complex at initial (0 ns) and end of simulation (100 ns). (**C**) RMSD of AKT-eriodictyol complex in MD analysis. (**D**) Simulate the conformation of AKT-eriodictyol complex at initial (0 ns) and end (150 ns). (**E**,**F**) RMSF of PI3K-eriodicityol and AKT-eriodicityol in MD analysis. (**G**,**H**) Rg of PI3K-eriodicityol and AKT-eriodicityol complexes in MD analysis. (**I**,**J**) Analysis of the number of hydrogen bonds in MD. These results represent three independent experiments.

Figure 9A reveals that the trajectory analysis of the PI3K-eriodictyol complex demonstrates a lack of substantial positional alteration in the ligand with respect to the protein throughout the entirety of the simulation. The binding of eriodictyol to PI3K remained consistently stable and minimal throughout the experimental duration. At 0 ns and 100 ns, the position of the ligand relative to the protein did not change significantly, indicating that the binding of PI3K-eriodictyol was stable (Figure 9B). According to the analysis of the movement trajectory of the AKT-eriodictyol complex (Figure 9C), the root-mean-square deviation (RMSD) value remained relatively stable within 20 ns of the simulated end segment, indicating that the protein-ligand complex had reached a relatively stable conformation and binding was relatively stable. The conformations of the protein-ligand complexes at 0 ns and 150 ns were extracted and displayed. It can be seen that there was a significant change in the pocket position of the ligand at 150 ns compared to 0 ns, while the binding pocket and position remained relatively close at 75 ns (Figure 9D). From Figure 9E–F, it is apparent that the overall Root-mean-square fluctuation (RMSF) of the PI3K-eriodictyol and AKT-eriodictyol complexes remains consistently low throughout the simulation, indicating the stability of the protein-ligand complexes. In Figure 9G,H, both PI3K and AKT exhibit the Radius of Gyration (Rg) values below 3.1 nm and 2.05 nm, respectively. Analysis of the trajectory of the protein-ligand complex’s motion suggests sustained stability in the binding between the ligand and protein throughout the entire simulation process. Furthermore, the fluctuations in the count of hydrogen bonds established between the proteins and ligands were assessed during the 100 ns simulation of PI3K-eriodictyol (Figure 9I). The findings demonstrate the presence of a considerable number of hydrogen bond interactions between the ligand and protein, indicating the enduring stability of the binding throughout the entirety of the simulation process. Similarly, the 150 ns simulation of AKT-eriodictyol (Figure 9J) revealed the maintenance of at least one hydrogen bond throughout the entire simulation process, further highlighting the stability of the complex binding. Overall, the combination of PI3K-eriodicityol and AKT-eriodicityol demonstrates relatively stable binding throughout the simulation process. The association between eriodictyol and PI3K and AKT has the potential to influence the protein’s conformation, consequently triggering downstream signal transduction pathways.

## 4. Discussion

ALI is an abrupt clinical syndrome that typically arises from viral infections, toxic substances, excessive alcohol intake, and drug overdose, and severe liver injury can result in failure of the liver and even death [18,19]. The pathogenic factors of ALI show certain geographical differences around the world, ALI is caused by acetaminophen in western developed countries, while viral hepatitis B-induced ALI is most prevalent in developing countries [20]. In recent years, due to drug abuse, the incidence rate of ALI has increased year by year in China; however, the existing liver-protective drugs cannot fully satisfy the clinical requirement. Hence, it holds immense importance to study medications for liver protection that have favorable outcomes and minimal adverse reactions. Natural compounds, renowned for their multitargeted actions and lower incidence of adverse effects compared to synthetic medications, have become the focus of modern anti-ALI research [21]. Consequently, an increasing number of researchers are directing their attention toward natural products for the prevention and treatment of liver injury. Eriodictyol is a hydroxylated flavonoid, and consuming a diet rich in flavonoid-rich foods is considered a potential functional approach to promote liver health and protect against ALI [22]. There are many common ALI models, among which LPS/D-GalN causes ALI similar to human viral hepatitis [23]. LPS/D-GalN-induced ALI has been extensively employed as a mouse model to explore liver-protective agents [24]. This model elicits a significant inflammatory response and facilitates hepatocyte apoptosis. D-GalN, known for its hepatotoxic properties, inhibits RNA and protein synthesis in hepatocytes. Furthermore, it induces hepatocyte apoptosis and necrosis by depleting uracil nucleotides [25], ultimately leading to acute hepatic failure. So this model was selected to do the in vivo anti-ALI test of eriodictyol.

The network pharmacology offers insights into the potential mechanism of eriodictyol in ALI by facilitating the prediction of the targeted distribution and pharmacological action of active compounds found in traditional Chinese medicine [26]. By leveraging the technological advancements and methodologies of network pharmacology, this research provides a predictive framework for understanding the potential mechanisms underlying the ALI-specific effects of eriodictyol. A total of 169 drug targets and 786 disease targets were obtained. Through the Venn diagram, we determine the 47 targets of eriodictyol related to ALI diseases. Through the analysis of the interaction between PPI proteins of anti-ALI therapeutic targets of eriodictyol, we found that eriodictyol may play an anti-ALI therapeutic role through core targets such as ALB, ACTB, AKT1, and VEGFA. The analysis of GO function and KEGG pathway showed that eriodictyol mainly passed through many biological processes, such as oxidative stress, and response to extracellular stimulation, mainly through PI3K/AKT, Fluid shear stress and atherosclerosis pathways to exert its function of liver protection. In order to further substantiate the mechanism of eriodictyol’s action, in vivo experiments were conducted to confirm the protective effect and elucidate the potential mechanism. The findings of this study indicated that eriodictyol exhibited a dose-dependent protective effect on ALI by decreasing ALT, AST, AKP, ALB, and γ-GT levels induced by LPS/D-GalN. Histopathological examination of liver tissue demonstrated that preventive treatment with eriodictyol significantly mitigated the LPS/D-GalN-induced liver histopathological changes.

BP analysis of GO function in network pharmacology showed that eriodictyol can act as an antioxidant [27]. MDA serves as the primary product of the peroxidation of polyunsaturated lipids, while SOD and reduced GSH are crucial enzymes involved in combating oxidative stress [28]. Our investigation demonstrated the induction of severe oxidative stress in mice by LPS/D-GalN, aligning with the research outcomes of Wang’s study [10]. Additionally, pretreatment with eriodictyol exhibits the ability to enhance the levels of the antioxidant GSH and SOD in the liver, while reducing the accumulation of MDA in hepatic tissue. These findings provided further evidence for the anti-oxidative stress capability of eriodictyol. The inflammatory response is another significant manifestation of LPS/D-GalN-induced liver injury. Eriodictyol pretreatment reduced the levels of TNF-α, IL-1β, and IL-6 in liver tissue, indicating its pronounced regulatory effect on the expression of inflammatory factors and its potent anti-inflammatory effect.

The PI3K-Akt-mTOR signaling pathway engaged in numerous cellular functional activities, including apoptosis, migration, and cellular invasion [29]. Anne-Katrien et al. found that when Toll-like receptor (TLR) mediated inflammation occurs, the PI3K signal will be inhibited, thus inhibiting the secretion of pro-inflammatory factors [30]. AKT, a significant downstream mediator of PI3K, plays a crucial role in cellular signaling [31]. In this study, KEGG enrichment analysis in the early network pharmacology showed that eriodictyol mainly resisted liver injury via the PI3K/AKT signaling pathway. The evaluation of PI3K and AKT expression in the liver tissue of mice across all experimental groups was conducted using the immunohistochemical technique in this study. Following eriodictyol treatment, there was an upregulation in the expression of PI3K and AKT in the liver tissue of mice. Consequently, to further explore this signaling pathway, we utilized PCR techniques to assess the expression levels of genes associated with it. Our findings indicated a downregulation in the mRNA expression of Pi3k, Akt1, and mTOR in liver samples from the model group. Conversely, eriodictyol demonstrated a dose-dependent reversal of this trend. Previous research has shown that Nrf2 activation is reliant on the PI3K/AKT pathway and that inhibiting PI3K leads to Nrf2 overexpression [32]. Therefore, the results indicated that eriodictyol can attenuate LPS/D-GalN-induced ALI by inhibiting oxidative stress and inflammation through the activation of the PI3K/AKT signaling pathway.

Apoptosis, a pivotal cellular process, is essential for maintaining the physiological balance of organs [33]. In our investigation, we observed an increase in TUNEL-positive hepatocyte apoptosis in the model group, while eriodictyol pretreatment significantly reduced the number of TUNEL-positive hepatocytes. These findings suggest that LPS/D-GalN can induce heightened apoptosis in liver tissue, whereas eriodictyol exhibited inhibitory effects on tissue cell apoptosis. The induction of liver tissue apoptosis by LPS/D-GalN was assessed using RT-PCR. In this study, the mRNA levels of Bax, Caspase-3, and Caspase-8 in the liver tissue of mice with LPS/D-GalN-induced liver injury were significantly elevated, whereas the mRNA level of Bcl-xl exhibited a significant decrease. However, eriodictyol pretreatment exhibited a dose-dependent reversal of these alterations. These findings support the notion that eriodictyol can inhibit tissue cell apoptosis, thereby alleviating LPS/D-GalN-induced ALI. Recent research has provided evidence that the activation of the PI3K and Akt pathways inhibits apoptosis [34], Consistent with our research findings, it can be concluded that eriodictyol possesses the ability to suppress oxidative stress, inflammation, and cellular apoptosis through the activation of the PI3K/Akt pathway, thereby mitigating ALI. Subsequently, the outcomes of molecular dynamics simulations authenticated the strong affinity of Eriodictyol towards PI3K and AKT, which has the potential to influence the conformation of PI3K and AKT, consequently impacting their downstream signal transduction pathways.

Nevertheless, it is important to acknowledge the limitations of our current study. While the PI3K/Akt pathway has been implicated in the influence of eriodictyol, it is essential to recognize that eriodictyol may exert its effects through additional pathways. A more comprehensive investigation is warranted to elucidate the precise mechanism by which eriodictyol operates in ALI. This study uses an animal model of ALI, but its protective effect on chronic liver injury needs further study. In conclusion, we have proved that eriodictyol has a significant liver protection effect in the experimental model in vivo and predicted PI3K/Akt as a possible signal pathway using network pharmacology and then verified it through the experiment in vivo. This substantiates the ability of eriodictyol to attenuate ALI by impeding oxidative stress, inflammation, and apoptosis through the activation of the PI3K/Akt pathway.

## 5. Conclusions

Collectively, we employed bioinformatics analysis techniques to assess the potential targets and underlying signal pathways implicated in eriodictyol’s therapeutic potential for ALI. Building upon this foundation, we established a mouse model of ALI induced by LPS/D-GalN to investigate the molecular mechanisms underlying eriodictyol’s hepatoprotective properties. The outcomes indicated that eriodictyol’s protective effects against ALI involve the activation of proteins within the PI3K/AKT signal pathways, regulation of oxidative stress, attenuation of inflammation, and inhibition of hepatocyte apoptosis. Therefore, we speculate that eriodictyol can be used as a natural liver protector to prevent liver injury.

## Figures and Tables

**Table 1:** Primers’ list.

Gene	Primer Sequence	
	Forward (5′-3′)	Reverse (5′-3′)
Pi3k	CCGTGATGGAAAATATGGCTT	AGCTAAAGACTCATTCCGGTA
Akt1	CGGTTCTTTGCCAACATCGT	CCTCATCGAAATACCTGGTGT
mTor	ATCCTGCACATTGACTTTGGG	ATGTGGTTCTGTAGTTGCCAT
Tsc2	CTGCCTCTGTTCATTATCACC	TTACGCATCAACTTCCAGCAA
Bax	ATGCGTCCACCAAGAAGC	CAGTTGAAGTTGCCATCAGC
Bcl-xl	TCGACTTTCTCTCCTACAAGC	GCCTCAGTCCTATTCTCTTCG
Caspase3	CTCTGGGATCTATCTGGACA	GATGACATTCCAGTGCTC
Caspase8	CTTCGAGCAACAGAACCACAC	TTCTTCACCGTAGCCATTCCC
Bid	GTTCATGAATGGCAGCCTGT	TGGAAGACATCACGGAGCAA
GAPDH	CATCCGTAAAGACCTCTATGCCAAC	ATGGAGCCACCGATCCACA

**Table 2:** The top 10 intersection targets.

Number	Name	Degree
1	ALB	84
2	ACTB	78
3	AKT1	78
4	VEGFA	68
5	HSP90AA1	64
6	HIF1A	62
7	PPARG	62
8	ESR1	60
9	SRC	58
10	PTGS2	54

## Data Availability

The data supporting this study’s findings are available upon request.

## Abbreviations

ALI: Acute liver injury; D-GalN: D-galactosamine; LPS: Lipopolysaccharide; ALT: Alanine aminotransferase; AST: Aspartate aminotransferase; AKP: Alkaline phosphatase; γ-GT: γ-glutamyltransferase; CHE: Cholinesterase; TP: Total proteins; ALB: Albumin; SOD: Superoxide dismutase; MDA: Malondialdehyde; GSH: Glutathione; IL-6: Interleukin-6; IL-1β: Interleukin-1 beta; TNF-α: Tumor necrosis factor α; PPI: Protein-protein interaction; GO: Gene ontology; KEGG: Kyoto encyclopedia of genes and genomes; DO: Disease ontology; H&E: Hematoxylin and eosin; CC: Cell component; BP: Biological process; MF: Molecular functions; TUNEL: TdT-mediated dUTP nick end labelling; RT-qPCR: Real-time quantitative polymerase chain reaction; MD: Molecular dynamics; RMSD: Root-mean-square deviation; RMSF: Root-mean-square fluctuation; Rg: Radius of gyration.
